# Effect of pay for performance to improve quality of maternal and child care in low- and middle-income countries: a systematic review

**DOI:** 10.1186/s12889-016-2982-4

**Published:** 2016-04-14

**Authors:** Ashis Das, Saji S. Gopalan, Daniel Chandramohan

**Affiliations:** London School of Hygiene and Tropical Medicine, London, UK; Health, Nutrition and Population Global Practice, The World Bank Group, Washington, DC USA

**Keywords:** Pay for performance, Quality of healthcare, Maternal and child health, Low- and middle income countries

## Abstract

**Background:**

Pay for Performance (P4P) mechanisms to health facilities and providers are currently being tested in several low- and middle-income countries (LMIC) to improve maternal and child health (MCH). This paper reviews the existing evidence on the effect of P4P program on quality of MCH care in LMICs.

**Methods:**

A systematic review of literature was conducted according to a registered protocol. MEDLINE, Web of Science, and Embase were searched using the key words maternal care, quality of care, ante natal care, emergency obstetric and neonatal care (EmONC) and child care. Of 4535 records retrieved, only eight papers met the inclusion criteria. Primary outcome of interest was quality of MCH disaggregated into structural quality, process quality and outcomes. Risk of bias across studies was assessed through a customized quality checklist.

**Results and discussion:**

There were four controlled before after intervention studies, three cluster randomized controlled trials and one case control with post-intervention comparison of P4P programs for MCH care in Burundi, Democratic Republic of Congo, Egypt, the Philippines, and Rwanda. There is some evidence of positive effect of P4P only on process quality of MCH. The effect of P4P on delivery, EmONC, post natal care and under-five child care were not evaluated in these studies. There is weak evidence for P4P’s positive effect on maternal and neonatal health outcomes and out-of-pocket expenses. P4P program had a few negative effects on structural quality.

**Conclusion:**

P4P is effective to improve process quality of ante natal care. However, further research is needed to understand P4P’s impact on MCH and their causal pathways in LMICs.

**Trial registration:**

PROSPERO registration number CRD42014013077.

## Background

Pay for performance (P4P) is an emerging health sector strategy to improve availability, quality and utilization of essential healthcare services. P4P aims at incentivizing performance of the providers and clients for uptake of key services and behavior changes [1]. P4P belongs to the category of innovative financing mechanisms that includes similar type of payment systems such as results based financing, performance-based financing, performance-based contracting, output-based aid, conditional cash transfer and cash on delivery [1]. In supply-side P4P, incentives are provided to achieve a pre-agreed set of results (outputs and outcomes) by improving the performance of health workforce and health facilities [2] and involves a strict monitoring of results in a stipulated time-frame. Typically, performance in a P4P program is measured through health outcomes, utilization of services, and quality of care [3]. Though P4P has been widely implemented in both high- and low-income settings, its primary focus and trajectory are context specific [3]. In low- and middle-income countries (LMICs), P4P is commonly used to achieve unmet millennium development goals (MDG) 4 and 5 on maternal and child health (MCH) [4]. However, the exiting knowledge on the effects of P4P is limited to utilization of a few services than quality of care [4]. Even in high-income countries, rigorous evidence on the effect of P4P on quality of care is limited [5–8].

Several LMICs in Asia and Africa have experimented P4P to improve MCH since 1990s, mainly to mitigate the burden of maternal and child conditions [4, 9, 10]. Clinical evidence indicates that quality of MCH care is a pre-requisite to reduce maternal and child mortalities [11]. An increased uptake of MCH services such as skilled birth attendance and newborn care without adequate quality cannot guarantee an improved MCH status [12]. Studies conducted in Cambodia, Democratic Republic of Congo, Burundi, Rwanda, Haiti have demonstrated improvements in maternal and child healthcare service utilization and to some extent better financial and management capacities with health facilities [13]. There is no synthesized evidence supporting the positive effect of P4P on quality of care. In addition, a systematic review conducted on P4P in LMICs asserts that the evidence is weak to conclude the impact of provider incentives on quality of care [4]. If P4P positively impacts only service utilization without corresponding improvements in quality of care, current investments on P4P in low-income countries may not be cost-effective to improve MDGs 4 and 5 [3]. In this systematic review, we assessed the effect of supply-side pay for performance on the quality of maternal and child health services in LMICs. While identifying the knowledge gaps in this area, we also explored the appropriateness of methods adopted by different studies to measure quality of MCH care under P4P.

### Defining quality of healthcare: definition and measures

Institute of Medicine (IOM) defines quality in healthcare as the “degree to which health services for individuals and populations increase the likelihood of desired health outcomes and are consistent with current professional knowledge” [14]. Donabedian describes healthcare service delivery as a continuum which includes structures, processes, and outcomes and asserts quality of care is an end product when the structures are translated to outcomes through the processes [15]. In the service delivery continuum, there is equal emphasis on each of the above mentioned aspects of quality. *Structural quality* consists of human and key material resources such as infrastructure, equipment, drugs and supplies, communication, and transport. Apart from having the needful material resources, it is also essential that they are put to practice to provide services. To deliver optimal quality of care, adequately skilled and motivated human resources should be available [15]. *Process* simply means whether services are provided optimally and safely following the standards of service delivery through technical and non-technical performance [15]. Technical performance entails delivering scientifically proven services at the appropriate time. For instance, during routine antenatal visit, a woman should undergo weighing; testing of blood and urine samples for infection and signs of pre-eclampsia; palpation of abdomen; and measurement of blood pressure and abdominal girth. Non-technical performance relates to interpersonal relationship, provider behavior, privacy, and confidentiality [13, 16–18]. Key consequences of the service delivery such as morbidity, mortality, out-of-pocket expenses, and client satisfaction constitute the *outcomes* [12]*.* In this review, we adopt Donabedian’s definition of quality in healthcare.

## Methods

### Protocol and registration

This study is registered with the PROSPERO international prospective register of systematic reviews (registration number CRD42014013077) [19]. A peer-reviewed protocol guided the conduct of review. This review is reported as per PRISMA guidelines [20].

### Selection of studies

Studies from low- and middle-income countries as defined by the World Bank income criteria were included [21]. Two of the authors (AD and SG) independently searched the literature, screened abstracts and retrieved full papers. Final selection of studies against the inclusion criteria was done independently by these authors and disagreements were resolved through a consultative process.

#### Inclusion criteria

1. Evaluation reporting results of any supply-side (i.e. facility/provider) P4P on a quantitative measure of MCH care quality
2. Study conducted in low- and middle-income countries
3. Published in English between January, 1990 and June, 2014. This selection is based on the fact that P4P programs started from the 1990s.
4. Presence of at least one comparison group
5. Study reporting statistical significance of the intervention than only descriptive analysis
6. Study meeting a minimum quality score of six, defined by two reviewers

#### Exclusion criteria

1. Study presenting the impact of P4P on only access to and usage of MCH care without any quality of care measures
2. Qualitative study or review
3. Study on P4P presenting non-MCH care
4. Study reporting only descriptive analysis
5. Study with a quality score of less than six

#### Type of studies

Studies were selected if they met the criteria used by the Cochrane Effective Practice and Organization of Care group (EPOC) [22]. The EPOC study designs are: randomized controlled trials (RCTs), clustered randomized controlled trials (c-RCT), controlled clinical trials (CCT), controlled before-after studies (CBAs) and quasi-experimental studies including interrupted time series. Given the dearth of literature on P4P and quality of MCH care, in addition studies having at least one intervention and one comparison group were included.

#### Types of participants

Study population comprised of women during pregnancy and post-partum period; children younger than five years; and health workers under assessment for a P4P program.

#### Type of interventions

P4P interventions in public or private sector, providing conditional financial incentives to facilities and/or providers to achieve certain performance measures on MCH services including quality were selected.

#### Operational definitions

Maternal health care included any routine or illness care received during the antenatal, delivery and postpartum period. Child health care included any care received from birth up to five years of age for any routine or illness conditions. Health workers were defined as medically trained personnel (doctors, clinical officers, midwives, and nurses) working at a primary or secondary care level in LMIC settings.

#### Outcomes of interest

Primary outcome of interest was quality of MCH disaggregated into structural quality, process quality and outcomes. Under structural quality, we considered availability of health facility infrastructure, skilled staff, equipment, commodities, and drugs. For process quality, we included adherence to standard protocols and guidelines for management of health conditions. Morbidity, mortality, out-of-pocket expenses for medical services in the healthcare facility, and client satisfaction constituted the outcomes.

### Information sources and search

Records were searched in several electronic search engines and databases namely MEDLINE, EMBASE, Global Health, PsycINFO, Econlit and Web of Science. Additionally, Google Scholar was searched electronically. Websites of key organizations involved in P4P programs (World Bank, DFID and NORAD) were purposively searched for published articles or working papers. A hand search enabled to retrieve certain relevant papers from the selected records. Contacts were made to authors and scholars in the field of P4P to identify additional studies.

The literature search was conducted during May-August 2014. Records published between January, 1990 and June, 2014 were selected. Each database had different search words as a combination of MeSH (medical subject heading) and non-MeSH terms using Boolean operators “AND” and “OR”. The search algorithm was developed based on a preliminary search in PubMed and Google Scholar. The thematic search words are given in Table 1.

**Table 1:** Search strategy

**Thematic Search**	
“provider performance” OR “provider incentives” OR “pay for performance” OR “performance-based financing” OR “performance-based incentives” OR “supply-side incentive” OR “provider performance” OR “results-based financing”	
AND	
“quality of care” OR “clinical standards” OR “structural quality” OR “process quality”	
AND	
“Maternal health/” [MeSH] OR “ante natal/pre natal” OR “post natal/postpartum” OR “child birth/delivery/institutionalized” OR “newborn/neonatal” OR “immunization/vaccination” OR “children/child” OR “nutrition/stunting/anemia”	
**Adjunct Search**	
“Developing countries/less developed nations/third world countries”[MeSH] OR “developing health Systems” [MeSH] OR Africa/sub-Saharan africa” [MeSH] “Central/south/latin america”[MeSH] OR “asia/central/south east Asia”[MeSH] OR “commonwealth of independent states”[MeSH] OR “indian ocean islands”[MeSH] OR “eastern europe”[MeSH] OR “south asia” OR “low income countries/low and middle income Countries”	

### Data items and extraction

Country and year of study, study settings and design, sample size, type of incentive (recipient, conditionality and frequency), comparison groups, outcome measures, and quality element of the outcome measures were extracted using a data extraction form. The primary author extracted the data and a second author validated the process.

### Summary measures and data synthesis

Where possible we presented either odds ratio or coefficient along with the confidence interval. Net effects of the interventions were calculated as the difference between intervention and control groups at baseline and follow up, and presented as percentage points, coefficients or absolute numbers in natural units. We considered an outcome statistically significant at 5 % level (*p* < 0.05). The reported outcomes were presented by the elements of quality, i.e. structure, process and outcomes. Due to heterogeneity of studies and presentation of results, no meta-analysis could be performed.

### Appraising methodological and reporting quality of included studies

We developed a customized quality assessment tool for appraising methodological quality of studies, adapted from Downs and Black [23]. The quality tool took into account methodological quality (randomization, baseline balance of key variables), external validity (representativeness of study sample, contamination of interventions), and reporting quality (clear description of objectives, interventions, outcomes, power calculations, findings). Our adaptation was reflected in scoring various types of studies with the highest score assigned to RCTs, replacing representativeness of patients with facilities, and removal of items related to blinding of randomization and patient adverse events. There were 18 quality indicators for RCTs and 17 for CBAs and each indicator had an indicative score. Wherever the description did not include a particular item mentioned in the quality assessment tool or it was unclear, we scored that item zero. Because of the variation in scoring between studies (i.e. RCT and CBA), we standardized the absolute scores to percentage to ensure comparability. Based on the aggregate quality score, studies were ranked as low (<34 %), moderate (34-66 %) and high (>66 %). Two of the authors (AD and SG) independently assessed the quality of studies, with any disagreements resolved through discussions.

## Results

### Study selection

**Fig. 1:**
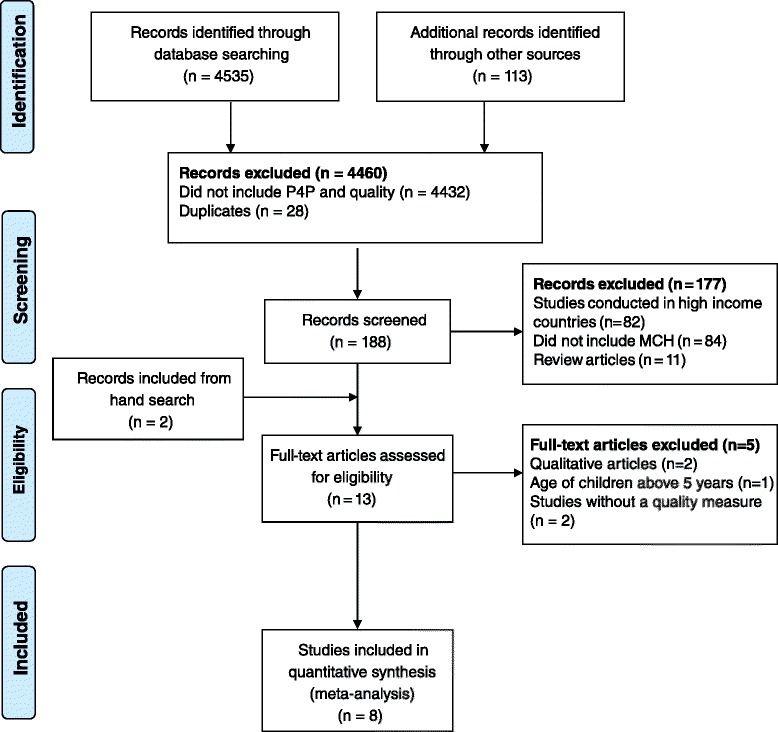
Flow diagram for selection of articles

Search from the databases identified 4535 records, and an additional 113 records were retrieved from other sources and personal communications with researchers. Screened records were 188 after removing duplicates and excluding records that did not mention P4P and quality. From 13 articles eligible for full-text assessment, only eight were included in the review. Details of the study selection are given in the Fig. 1.

### Study characteristics and settings

There were four CBAs, three cluster RCT and one case control with post-intervention comparison [24–31] of P4P programs on MCH care in Burundi, Democratic Republic of Congo (DRC), Egypt, the Philippines, and Rwanda (Table 2). Five studies took place in primary care health centers [24–27, 31], two reported results from district level hospitals [28, 29] and one was conducted in both primary and secondary level facilities [30].

**Table 2:** Study characteristics and Quality score

Author, year; Country	Study Design	Program setting	Incentives			Comparison Group	Outcome measures	Quality element	Methodological Quality score (%)
			Recipient	Conditionality	Frequency				
Peabody et al., 2011; Philippines [28]	CRT	30 District hospitals (DH)	Providers	Physician competence score, case load and patient satisfaction	Quarterly	DHs from matched districts without P4P	Quality of care, utilization of services of children under-five	Process quality	13(72)
Peabody et al., 2014; Philippines [29]	CRT	30 District hospitals	Providers	Physician competence score, case load and patient satisfaction	Quarterly	DHs from matched districts without P4P	Quality of care, utilization of services of children under-five	Clinical outcomes for under-five children	14(78)
Huillery and Seban 2014; DRC [30]	CRT	152 Facilities (primary and secondary level)	Facilities	Utilization of services	Monthly	Facilities in control districts receiving equivalent fixed payment	User fees, service accessibility, service quality and utilization, population health status, health facility revenue, health workers’ satisfaction, anxiety, motivation	Patient perceived quality and structural quality	14(78)
Basinga et al., 2011; Rwanda [27]	Controlled before and after	Rural health centers - 80 in intervention and 86 in control	Facilities	Utilization of 14 key MCH services and quality of services delivery	Quarterly	Facilities under input-based financing received funds equivalent to P4P payments	Prenatal visits, institutional delivery, quality of ANC, child preventive care visits and immunization	Process quality of ANC	15(88)
Bonfrer et al., 2014; Burundi [25]	Controlled before and after	700 facilities	Facilities	Obtaining quality and quantity of services	Monthly for quantity and quarterly for quality	Households in the provinces where P4P was not implemented	Utilization and quality of MCH services	Process quality of ANC	10(59)
Bonfrer et al., 2014; Burundi [26]	Controlled before and after	700 facilities	Facilities	Obtaining quality and quantity of services	Monthly for quantity and quarterly for quality	Facilities in control districts receiving normal input financing and salary bonus	Maternal and under-five services	Structural and process quality	9(53)
Soeters et al 2011; DRC [24]	Controlled before and after	Two districts	Facilities	Utilization of services	Monthly for quantity and quarterly for quality	Two control districts with characteristics similar to intervention districts receiving essential drugs, equipment and fixed staff performance bonuses	Not mentioned	Patient perceived quality, structural and process quality	7(41)
Huntington et al., 2010; Egypt [31]	Case control post-test only	Primary health centers	Providers	Quantity and quality of preventive, curative and quality of MCH services	Monthly	Primary care providers in control arms got flat rate salary supplements	Quality of ANC, child care services and family planning care	Process quality of ANC, family planning and child care	7(41)

Two cluster randomized trials of performance based salary bonus to health care providers were reported from the Philippines [28, 29]. In these two RCTs, 30 district hospitals from districts matched by socio-economic, demographic and health profile were randomized to one of the two intervention arms or to the control arm. In the cluster randomized trial in DRC, 96 health facilities in one district were randomly assigned to intervention and control arms [30]; the intervention arm received performance-based incentives, while control arms only input-based financing.

The CBA in Rwanda randomly assigned 80 health facilities from 12 districts to receive a P4P intervention and 86 health facilities from seven districts to receive an equivalent input-based financing [27]. The CBA of a P4P program in DRC [24] allocated two districts to receive performance-based incentives and compared the outcomes with another two districts having similar socio-economic characteristics. From Egypt, a post-test only comparison study that assessed a P4P program in primary health centers receiving incentives for more than two years [31] was reported. Comparison groups received equivalent additional incentives as salary top-off without any performance conditionality. Two studies of P4P program were reported from Burundi. One study used a CBA for the pilot phase [26] and the second study compared population level outcomes on quality of antenatal care between P4P and non-P4P provinces in the nation-wide roll-out phase [25].

### Characteristics of performance measures and payments on quality of care

Studies described diverse performance measurement and payment mechanisms for quality of care. Performance mechanisms included achieving a certain level of volume and quality of MCH services. Three programs incentivized quality of care with limited set of indices [26–29, 32]. Three others utilized a composite index including availability of human and material resources, compliance to national standards, proper record keeping, and client satisfaction [24, 30, 31].

Payment systems included payment for individual health workers [28, 29, 31] or for facilities [24–27]. Incentives accounted for 5 % of physician’s salary in the Philippines [29] and 275 % times the base salary of a primary health facility staff in Egypt [31]. In DRC, the monthly payments to facilities ranged from $200 to $4000 [24].

In DRC, apart from incentivizing utilization of MCH services, the program offered a bonus of up to 15 % of the subsidies to facilities on quality of care [24]. Performance indicators in Egypt consisted of preventive, curative and quality of care measures (completeness of medical records, patient satisfaction and waiting time) on MCH [31]. The Rwandan program incentivized facilities on a combination of service volume and quality for MCH [27]. The P4P facilities in the Philippines received incentives linked with the average clinical competence scores of physicians, facility caseload, and average utilization of services (quantity) and adherence to national standards and protocols (quality) [28]. This adherence to treatment guidelines was assessed for vaccinations, family planning, tuberculosis, HIV and antenatal care.

### Reporting of quality of care in studies

Studies adopted different methods to report quality of care outcomes. Generally, quality was reported either objectively (direct observation of availability and receipt of services as per national standards of care) or reported by patients (e.g. receipt of services, perceptions on staff attitude, waiting time, quality of services). Means of verification of quality were through exit and household interviews (patient perception and experiences), review of records, direct observation (infrastructure, drugs and equipment) and vignettes. Six studies utilized household interviews to measure quality [24–27, 29, 30], while two studies each employed exit interviews [30, 31], review of records [24, 26], and direct observations [24, 26]. Only one study applied vignettes as a means of verification [28].

### Risk of bias across studies

The mean quality of studies score was 63.8 % with a range of 41 to 88 %. Two RCTs were of high quality with a score of 78 % [29, 30] and one RCT had a score of 72 % [28] (Table 2). Among the five CBA studies, only one [27] was of high quality, scoring 88 %. Two studies were of medium quality with a score of 53 and 59 % [25, 26]. Two CBA studies were of low quality with a score of 41 % [24, 31].

Five studies did not report baseline participant characteristics, representativeness of the participants or facilities, estimates of random variability and actual probability values [24–26, 30, 31]. Three CBAs did not mention the matching criteria for control and intervention sites [24, 27, 32]. Studies with selection bias did not consider the use of instrumental variable techniques to identifying treatment effects. Seasonality might have confounded the outcomes in the DRC study as the surveys were conducted at two different seasons [24].

### Effects of interventions

#### Structural quality

Studies gathered results on structural quality from direct observation and review of records. Four studies described the effect of P4P on elements of structural quality (Table 3). The availability of qualified staff increased by 15 % points and patient perceived availability of drugs improved by 37 % points in DRC [24] compared to pre-intervention period. In the Philippines, P4P improved physicians’ knowledge to manage under-five diarrhea and pneumonia (coefficient 1.6; *p* < 0.001) [29]. However, another study showed some negative effects of P4P on structural quality in DRC [30]. These negative effects were observed on overall structural quality index and availability of drugs and vaccines in the facility [30]. Patient perceived availability of drugs decreased (coefficient -308.33; *p* < 0.001). There was a decline in structural quality index based on interviewers’ observation (coefficient -0.525; *p* = 0.014), equipment index (coefficient -0.639; *p* = 0.026) and vaccine availability (coefficient -0.744; *p* = 0.034). In this study, P4P did not show any effect on patient perceived equipment quality, infrastructure index and the number of types of drug currently available. Patient perceived availability of drugs in Burundi (coefficient 0.04; *p* = 0.492) and equipment quality in DRC (coefficient 0; *p* = 0.997) did not change under the P4P program [26, 30].

**Table 3:** Effect on structural quality

Variable	Net treatment effect	*P* value
Qualified staff in facilities^a^	15	<0.05
Sufficient drug availability (patient perceived)^b^	0.04	0.492
Provider clinical knowledge on child health (Mean Vignette score)^c^	1.6	<0.001
Patient perceived availability of drugs (%)^a^	37	<0.001
Patient perceived equipment quality^d^	0	0.997
Structural quality index based on interviewers’ observation^d^	−0.525	0.014
Infrastructure index^d^	0.184	0.372
Equipment index^d^	−0.639	0.026
Number of types of vaccine currently available^d^	−0.744	0.034
Number of types of drug currently available^d^	0.236	0.646

^a^Soeters et al. [24]; ^b^Bonfrer et al. [25]; ^c^Peabody et al. [28]; ^d^Huillery and Seban [30]

### Process quality

Studies reported process quality results from direct observation and review of records. Four studies presented P4P’s impact on various elements of process quality (Table 4). One study reported P4P’s effect on history taking and examination of pregnant women during ANC [31]. Two studies reported the effect on prescription and treatment of pregnant women and under-five children [30, 31]. Three studies mentioned about patient reported process quality on MCH services [29, 32, 33]. The study in Egypt showed the P4P increased the chances of a provider asking about parity (coefficient 11.4; *p* < 0.05) and past illness (coefficient 16.4; *p* < 0.01) during ANC visits [31]. But, the P4P did not significantly influence the chance of a provider enquiring about a pregnant women’s name, age and last menstrual cycle. In this study, P4P increased likelihood of measuring blood pressure (coefficient 8.4; *p* < 0.01), testing blood (coefficient 12; *p* < 0.01) and urine (coefficient 20; *p* < 0.01) during ANC visits. However, P4P program did not influence the chances of being weighed or fetal heart rate checked. The P4P increased provider’s adherence to explaining medicine intake for children under five years (coefficient 11.1; *p* < 0.05), follow-up treatment (coefficient 24.2; *p* < 0.05) and medicines (coefficient 10.5; *p* < 0.05). Yet, the program could not improve provider practices on treatment, prescribing iron, injections, vitamins, and tetanus toxoid for ANC visits. In Rwanda, P4P increased the ANC quality index in primary health centers (coefficient 0.157; *p* = 0.02) [27]. In DRC, P4P improved patient’s perceived quality of care index (coefficient 15; *p* < 0.05) and professional quality score of facilities on MCH services (coefficient 26; *P* < 0.001) [24]. Patient perceived overall quality of care index is the aggregate score given by the patient for various dimensions of provider behavior and competence (e.g. how provider explores the case scenario, how provider explains the health condition, how much respect a provider gives for the patient etc.) Professional quality score of the facility is a composite score consisting of structural and process elements as shown by both studies in DRC and Burundi. However, P4P program did not influence provider’s adherence to standardized medical procedure for any MCH service (coefficient -0.015; *p* = 0.695) [30].

**Table 4:** Effect on process quality

Variable	Net treatment effect	*P* value
History taking		
Asked name during ANC visit^a^	4.3	NS
Asked age during ANC visit^a^	4.5	NS
Asked parity during ANC visit^a^	11.4	<0.01
Asked date of last menses during ANC visit^a^	2.9	NS
Asked past illnesses during ANC visit^a^	16.4	<0.01
Examination		
Examined weight during ANC visit^a^	5.8	NS
Examined blood pressure during ANC visit^a^	8.4	<0.01
Examined fetal heart rate during ANC visit^a^	10.9	NS
Prescription and Treatment		
Asked for blood test during ANC visit^a^	12	<0.01
Asked for urine analysis during ANC visit^a^	20	<0.01
Explained intake of tetanus toxoid during ANC visit^a^	−8.4	NS
Explained intake of Vitamins during ANC visit^a^	5	NS
Explained medicine intake for under-five (%)^a^	11.1	*p* < 0.05
Overall treatment procedures (patient perceived) during ANC visit^a^	−5.5	NS
Drugs prescribed to pregnant women without examination^b^	0.02	0.66
Children received injection (%)^a^	−6	*p* < 0.05
Children received follow up (%)^a^	2.4	*p* < 0.05
Children given medicine (%)^a^	24.2	*p* < 0.05
Overall process quality		
Compliance rate with medical procedure, any service^b^	−0.015	0.695
ANC process quality score^c^	0.157	<0.001

*NS* Not significant

^a^Huntington et al. [31]; ^b^Huillery and Seban [30]; ^c^Basinga et al., [27]

### Quality outcomes

Studies obtained results on quality outcomes from review of records, exit interviews, household interviews and vignettes. Five studies demonstrated the effect of P4P on patient knowledge on managing health conditions, morbidity, mortality, out-of-pocket expense and client satisfaction (Table 5) [24, 29–32].

**Table 5:** Effect on quality outcomes

Variable	Net treatment effect	*P* value
Patient knowledge		
Women knew medicine-use in prenatal period^a^	12	<0.05
Women knew medicine-use in prenatal period^b^	−0.072	0.039
Health outcomes		
CRP negative for under-five^c^	0.84	0.497
Not anemic under-five^c^	−4.87	0.253
Good GSRH for under-five^c^	7.37	0.001
Weight-for-height z-score of under-five^b^	−0.347	0.306
Number of under-five deaths last year in households^b^	12	0.55
Out-of-pocket expenses		
Fee paid for the delivery^b^	301.24	0.762
Fee paid for the last postnatal visit^b^	−71.637	0.35
Fee paid for the last prenatal visit^b^	−112.969	0.125
Fee paid for the last immunization shot^b^	−22.096	0.237
Cost of drugs purchased by the patient at facilities^b^	−1106.16	0.005
Client Satisfaction		
Felt cured (pp)^d^	11	NS
Acceptable waiting time (pp)^d^	7	NS
Respect by staff (pp)^d^	12	<0.10
Felt cured (coefficient)^e^	0.09	0.012
Waiting time reasonable (coefficient)^e^	−0.12	0.318
Personnel respectful (coefficient)^e^	−0.02	0.718
Adequate consultation time (minutes)^b^	1.028	0.422
Pregnant women satisfied on user fees^b^	0.012	0.48
Pregnant women satisfied on welcome quality^b^	−0.027	0.442
Pregnant women dissatisfied on user fees^b^	0	0
Pregnant women dissatisfied on welcome quality^b^	0	0.946
Pregnant women satisfied on total care quality^b^	−0.005	0.671
Patient overall satisfied^b^	0.013	0.359
Overall quality of care		
Overall patient perceived quality score on ANC (pp)^d^	25	<0.05
Overall professional quality score of health centers (pp)^d^	26	<0.001
Total facility quality score (coefficient)^e^	17.24	0.062
Patient perceived quality of care (coefficient)^e^	0	0.924

*NS* Not significant

^a^Huntington et al. [31]; ^b^Huillery and Seban [30]; ^c^Peabody et al., [29]; ^d^Soeters et al [24]; ^e^Bonfrer et al. [25]

#### Patient knowledge

Number of pregnant women knowing the usage of pre-natal drugs increased in Egypt (coefficient 12; *p* < 0.05), while patient’s knowledge on drug intake decreased in DRC (coefficient -0.072; *p* = 0.039) [30, 31].

#### Health outcomes

In the Philippines, there was a small improvement in patient reported health measure for under-five (coefficient 7.37; *p* = 0.001) [29]. However, P4P had no effect on the prognosis of acute infections or on the incidence of anemia among sick children after 6 to 10 weeks of discharge from hospital. Under-five children did not experience any improvements in their weight-for-height z-score (coefficient -0.347; *p* = 0.306), longevity (coefficient -0.012; *p* = 0.55) or infants’ survival (coefficient -0.01; *p* = 0.093) in the DRC program [30].

#### Out-of-pocket expenses

In DRC [30], P4P reduced patient out-off-pocket expenses on purchase of drugs at facilities (coefficient -1106.16, *p* = 0.005). On the contrary, there was no significant effect on fee paid for immunization, delivery and ANC and PNC visits.

#### Client satisfaction

Three studies reported how P4P could influence patient satisfaction on consultation time, provider behavior, waiting time, user fee, welcome quality, overall quality of care and cure. In the DRC program, no improvement on provider attitude towards patients (coefficient 12; *p* <0.10) [24] was observed. Patients’ chance of feeling cured was higher under P4P program in Burundi (coefficient 0.09; *p* = 0.012) [26]. The DRC P4P program did not affect level of client satisfaction on adequacy of consultation time, overall quality of care, user fee and welcome quality [30].

#### Overall quality of care

Two studies demonstrated the effect of P4P on overall quality of care of MCH services considering structure, process and outcome measures (Table 5). The P4P program in DRC could enhance the total professional quality score of health centers (coefficient 26; *p* < 0.001) and patient perceived overall quality index (coefficient 25; *p* < 0.05) [24]. However, the facility quality score in Burundi though improved, it was not statistically significant (coefficient 17.24; *p* =0.062) [26].

## Discussion

### Summary of evidence

This systematic review makes a unique attempt to examine the effect of P4P on multiple quality of care elements by using a defined framework. This review found that the current evidence indicating P4P’s effect on quality of MCH in LMICs is skewed towards process quality and antenatal care. Feeble evidence showing P4P’s impact on quality of MCH care was mainly due to three reasons; 1) program evaluations did not adequately explore quality of care; 2) evaluations were mostly not powered enough to examine quality elements; and 3) P4P could not affect quality of care to a large extent.

The positive effect of P4P was observed only on limited aspects of MCH quality elements. Studies focused predominantly on antenatal care than delivery, EmONC, post natal care and under-five child care. Strength of evidence on maternal and neonatal health outcomes and out-of-pocket expenses was also limited. P4P program fetched a few negative outcomes on structural quality such as a reduction in the level of availability of drugs and equipment.

Despite targeting to improve structural quality of facilities, P4P programs could only improve availability of skilled staff, drugs and provider’s clinical knowledge. On the contrary, P4P negatively affected availability of equipment and vaccines [30]. The evidence was considerably positive on provider adherence to treatment protocols on ANC and child care. Patient out-of-pocket expenses on MCH did not reduce considerably under P4P programs, though out-of-pocket costs on drugs were reduced. However, client satisfaction did not substantially improve under P4P.

### Implications for policy and research

As P4P programs intend to reduce maternal and child deaths in LMICs, it is essential to demonstrate their potential to improve quality of MCH care comprehensively than process quality alone. Improving provider adherence to treatment guidelines on ANC alone cannot guarantee an improved maternal and child health. Clinical evidence suggests that quality of skilled birth attendance, EmONC and post natal care are necessary to reduce maternal and neonatal deaths [11]. There could be a possibility of insufficient provider skills affecting the process quality on delivery, EmONC and post natal care [11]. Thus, adequate attention should be given to evaluate the evidence on other aspects of MCH care.

Ensuring structural quality such as facility infrastructure, equipment, drugs and supplies is equally pertinent to offer quality care on MCH [11]. Absence of these minimum standards can affect patient satisfaction and in turn reduce demand for services in the long term [11]. Inadequate structural quality could be a reason for the poor client satisfaction in the studies [11]. Several P4P programs provided autonomy and funds to facilities to enhance structural quality. P4P programs in Egypt, Burundi, Rwanda and DRC also had routinely monitored structural quality. However, the prevailing positive evidence on structural quality is minimal. The DRC program faced negative effects on structural quality index, as facilities could not spend on infrastructure and equipment due to their reduced revenue under P4P. The adequacy of funds for infrastructural innovations under P4P programs needs to be investigated. Evaluations of P4P programs in high-income settings reflect that proportion of facility revenue is significant to improve quality of care [6].

There could be also a possibility of limited motivation and capacity among health workers and managers restricting innovations on strengthening structural quality, as evident in many LMICs [30]. Currently, it is unknown if the prevailing complex procurement system and managerial bottlenecks in the service delivery system to improve structural quality are better under P4P programs. Otherwise, these inefficiencies could retard structural quality improvement under P4P [30]. In addition, if there was no incentive for structural quality improvement, it could have been neglected with a preference for other incentivized indicators (known as cherry picking) [30].

Studies reflect that providers are motivated to improve process quality of care by adhering to treatment guidelines. There could be numerous reasons for their elevated motivation such as financial incentives, regular supervision, patient feedback and improved facility functioning [33]. Several demand-side financing programs proved that increased patient load negatively affects provider efficiency to handle high volume of patients and this could potentially reduce process quality of care in due course of time [33]. Thus, specific attention to retain process quality under P4P programs through optimum provider-patient ratio is needed.

Some P4P programs did not intend to charge user fee, but patient out-of-pocket expense was not reported to be lesser under P4P. Additional research is needed to explore specific cost drivers for out-of-pocket expenses under P4P. In DRC, facilities could not offset the revenue loss from reduced user fee as there was not sufficient demand generation, negatively affecting quality of care [30]. Design of P4P programs need to approach the issue of utilization and quality of services comprehensively by addressing both demand- and supply-side challenges.

Partial positive effect of P4P on quality of MCH care asks for a deeper investigation into role of design, implementation and evaluation of P4P programs. According to a review of P4P in high income countries, quality of care is the final outcome of the changes brought in by incentives at provider level, provider group level and health system level [6]. However, P4P programs in LMICs do not provide any similar evidence. Further, there could also be a possibility of preferential attention to P4P services at the cost of non-incentivized services or positive spillover from incentivized to non-incentivized services [8, 34]. None of the studies reported effects on non-P4P services.

Morbidity and mortality are difficult to attribute to the quality of care delivered because various factors such as severity and pre-existing illnesses, delayed care seeking, and non-adherence to treatment would affect these outcomes [15]. Since most of the P4P programs were less than two years, their evaluations did not potentially have adequate statistical power to explore the impact on mortality. Also, results need to be interpreted considering contextual factors e.g. duration and design of interventions, size of incentives, frequency of payment, timing of evaluation, representativeness of intervention areas and presence of private providers. Study sites were small and in some, were not representative of the country. Effectiveness can be different if intervention is exclusively on quality than many performance targets as shown in the studies.

A few studies with CBAs utilized matching of administrative areas such as provinces or districts [24, 30]. However, within these administrative areas there were gross heterogeneity among facilities in terms of infrastructure, staffing, catchment population and service volume. These differences can be minimized during the analysis if further matching of facilities could be performed. Matching technique such as propensity score matching which allows for comparison of effects between similar units can be tried to strengthen the rigor of evidence [35]. Studies in Rwanda, Egypt and DRC have tried to balance the financial resource effect across intervention arms by providing equivalent financing [27, 30, 31]. However, none had attempted to balance the effects of additional supportive interventions such as supportive supervision, continuous quality measurement, consistent use of checklists and operational plans that might have led to overestimation of P4P’s effects.

### Limitations

Most studies focused on other performance indicators such as service usage along with quality of care, restricting an in-depth exploration on the latter. This review may suffer from publication bias, as the existing literature may predominantly include studies reporting positive results. Despite these limitations, this review has attempted to include various quality of care elements comprehensively and explored in-depth.

## Conclusions

This systematic review showed that P4P is effective to improve process quality of ante natal care but not so effective on improving structural quality, customer satisfaction, out-of-pocket expenses and maternal and child health status. Several studies neither explored the effect of P4P on quality of MCH in-depth, nor were powered enough statistically. Further research is needed to understand P4P’s impact on EmONC, delivery and post natal care and their causal pathways in LMICs.
